# Explicating Child-Driven Patterns of Parent-Child Responsivity in Fragile Families: A Longitudinal Approach

**DOI:** 10.3389/fped.2022.813486

**Published:** 2022-03-15

**Authors:** Jessica Blume, SuJung Park, Miranda Cox, Ann M. Mastergeorge

**Affiliations:** Department of Human Development and Family Sciences, Texas Tech University, Lubbock, TX, United States

**Keywords:** child-driven effects, pro-social behavior, receptive language, parenting qualities, longitudinal, Fragile Families

## Abstract

It has been well-established that development occurs in the context of a transactional framework, with bidirectional parent-child interactions influencing both proximal and distal outcomes. In particular, child vocabulary development is sensitive to parenting qualities including warmth, sensitivity, and control as well as parental stimulation including language input and access to learning enrichment activities. Similarly, these parenting qualities are influenced by and influence children's development of pro-social behaviors. Given the foundational role of both language and pro-social skills for academic achievement and the establishment of healthy relationships across the lifespan, a comprehensive understanding of the magnitude, stability, and reciprocity of such interactions across childhood has the potential to better inform early intervention and prevention practices and highlight risk and resilience factors. This study investigated the concurrent and successive transactional relationships between child pro-social behavior, child emergent language, and parenting qualities within a large, longitudinal sample. This study utilized Waves 3, 4, and 5 of the Fragile Families and Child Well Being Study (FFCWBS), corresponding to focal child age 3, 5, and 9 years, respectively. A series of Structural Equation Modeling (SEM) with full-information likelihood (FIML) estimation (*n* = 3,422) including child prosocial behavior, receptive vocabulary, and supportive parenting behaviors was tested and compared. Our findings indicate significant, positive associations over time between child pro-social behavior and receptive vocabulary, and parenting quality across all three stages of early child development. The steady decline in magnitude of these associations over time highlights the importance of synergistic parent-child interactions in toddlerhood as an early opportunity to propel these developmental outcomes and supportive parenting behaviors. Patterns of change in child pro-social behavior skills and parenting qualities remained positive and relatively stable, while observed growth in child receptive vocabulary skills increased in magnitude over time. Additional investigation of indirect effects specified the role of receptive vocabulary, as well as the bolstering role of prosocial behavior, in eliciting responsive parenting qualities over time.

## Introduction

Early and middle childhood are crucial periods of growth in a variety of cognitive and social-emotional domains. Across child development, pro-social behavior and child language remain relatively stable over time (1–4). And, while both of these skills are fostered early in the context of parent-child interactions, acquisition of these skills also paves the way for children to further develop and refine these skills while socializing with peers. Parenting styles and behaviors also remain relatively stable and influence how children communicate with others and navigate social situations (5, 6). In the transition from early childhood to middle childhood, children become more independent and interact with others more frequently in a variety of social contexts (7, 8). Further, child characteristics such as temperament and emotion regulation can shape patterns of parental responsivity (9–12). The transactional nature of parent-child relationships has been well-established, particularly within the early and middle childhood periods.

Children are at higher risk for a variety of detrimental short-term and long-term outcomes including poor language skills at school entry, more severe behavior problems, and greater difficulty with social competence when raised in families facing socioeconomic adversity (13–15). These risks are exacerbated by differences in parent-child interaction characteristics as parents raising a child in poverty are more susceptible to stress, depression, discrimination, violence, and trauma, which can impede parent responsivity and sensitivity to their children (16, 17). And, parents from low-income backgrounds generally spend less time engaging with their children through cognitive stimulation opportunities at home such as playing and reading together (18–20). Further, reduced opportunities for social-emotional learning and positive engagement at home intensify the developmental risks associated with poverty status, while more frequent reciprocal engagement routines can actually provide buffering effects (21–23).

Given the important role of both pro-social skills and language for life course pathways, knowledge of the magnitude, stability, and reciprocity of parent-child interactions across childhood for children at risk due to poverty status (e.g., “fragile” families) may contribute to more efficient early intervention and prevention practices and enhanced understanding of risk and resilience factors. Thus, this study investigated the concurrent and successive transactional associations between child pro-social behavior, and child receptive language, and supportive parenting across early and middle childhood.

### Parallels Between Child Pro-social Behavior and Receptive Language

For most children, spoken language production begins close to the first birthday (24, 25). And, during toddlerhood many children begin to produce word combinations and a rapid period of growth in spoken language production and processing occurs as children approach preschool age (26, 27). Similarly, pro-social skills such as the inclination to help others emerge during toddlerhood and become routine behaviors as children develop social competence (28, 29). Thus, early social behavior and language skills are intertwined, and both are vital for the transition to primary school and subsequently middle childhood (30–32).

Difficulty with either skill, and in particular difficulty with both skills, contributes to behavior problems which can lead to poor development of friendships and struggles with peer acceptance (6, 33). Routine synchronous social exchanges with parents, teachers, and peers provide opportunities to hone other crucial skills like self-regulation and problem-solving (10, 34, 35). And, for children experiencing socioeconomic disadvantage, social skill strengths have been identified as a mediator in the association between language difficulties and behavioral problems (36). Both pro-social behavior and language skills are predictive of later academic achievement outcomes, thereby influencing attainment of higher education goals, as well as development of stable romantic relationships in young adulthood (37, 38). That is, these skills combined play a pivotal role in positive life course trajectories.

### The Influential Role of Parenting Behavior Qualities in Child Development

Parenting behavior plays an instrumental role in shaping both child social competence and language development. When children approach preschool age, social skills and language become more reciprocal and integrated; more socially competent children engage in more language exchange opportunities while children who have received limited language input at home from caregivers will engage in fewer social interactions, thereby experiencing fewer opportunities to improve social competence (1, 39). For instance, Bratsch-Hines et al. (40) evaluated this pathway longitudinally: more positive parent-child verbal interactions contributed to stronger language skills at child age 3 years as well as stronger child social skills in kindergarten. While Barnett et al. (1) identified similar associations between sensitive, positive parenting, language gains, and social competence gains during toddlerhood, most work to date has focused on growth in either language (41, 42) or social competence (43, 44), not both child development outcomes. Additionally, language gains have also been observed in low-income samples when sensitive parenting and positive parent-child interactions are an early intervention target (34, 45). And, adverse outcomes including child aggression and disordered language disorders have been observed when harsh parenting strategies including spanking are utilized (33, 43). Previous work has noted parenting behaviors and parenting stress as two of the most prominent contributing factors for child development outcomes, and when parents are raising families in the context of poverty they are more likely to be exposed to stressors such as neighborhood-related fear (46, 47). These family-level socioeconomic risk factors can contribute to adverse parent practices, but these risk effects may be mitigated with supportive parenting practices such as maternal warmth and parental monitoring (48, 49).

Among the specific parenting behaviors linked to both child social skills and language growth, supportive and responsive parenting has continued to gain empirical ground. For instance, a large-scale study (*n* = 1,364) investigated the stability of parenting behaviors across early development (e.g., child age 0–6 years), adverse parenting behaviors like hostility and negative regard were not regularly noted over time while parenting qualities like sensitivity, supportiveness, and stimulation were highly stable over this period (50). Additional evidence has confirmed stability of sensitive parenting practices, primarily focused on parenting practices during infancy and toddlerhood (1, 51). Also labeled facilitative parenting, parenting characterized by warmth and sensitivity has been found to contribute to child pro-social behavior (52, 53), above and beyond dysfunctional parenting (54). Supportive and responsive parenting behaviors have also been established as facilitators of cognitive growth (55), particularly language growth (56, 57). To date, however, studies focused on describing patterns of change in parenting behaviors, child social skills, and child language are often limited to changes within a specific developmental period such as infancy or late childhood; rarely have studies investigated these associations with a large, nationally representative sample across multiple child development stages.

### Reciprocal Patterns in Parent-Child Engagement

While parenting behaviors shape child behavior, children also elicit changes in parental wellbeing and responsivity; these child-driven effects are especially prevalent during developmental transition periods such as toddlerhood (58) and middle childhood (59, 60). Reciprocal associations are evident across child development but shifts in magnitude and direction of effects occur in these transition periods (10, 61). For instance, early parenting practices like warmth shape child cognitive schemas, and lack of parental warmth has been linked to child to parent aggression transmission (62). As parents provide developmental support, they also react to individual child characteristics such as temperament, intelligibility, attentional focus, activity level, and personality (63, 64), and this transactional feedback loop continues as children subsequently react to parent behaviors (65). Child characteristics and behavior may also influence parent stress levels, potentially exacerbating stress parents are already experiencing due to environmental risk factors (i.e., single parent status, financial insecurity, and depression) and eliciting shifts or fluctuations in parenting style (66, 67). Upon evaluation of reciprocal relations between positive parenting and child pro-social behavior in a large global study, higher levels of child pro-social behavior in late childhood elicited greater subsequent parental warmth and involvement while reciprocal relations in the inverse direction were non-significant (68). Additional studies that have evaluated reciprocal parent-child relations across more broad developmental periods (e.g., child age 2–7 and 8–13 years) have specifically identified child-driven effects, such that child behavioral patterns have enacted a robust and sustained influence on parenting practices (69, 70). But, much of the empirical work to date has focused on the reciprocal relations between negative parenting practices and adverse child outcomes [e.g., (71, 72)], Thus, further investigation of reciprocal effects specific to parenting practices and child factors that interact synergistically throughout the transition from early to late childhood is needed.

### Current Study

The present study utilized data from the Fragile Families and Child Wellbeing Study (FFCWS) to investigate developmental trajectories of child pro-social skills and receptive vocabulary, as well as supportive parenting, with FFCWS sample data from Waves 3, 4, and 5 which reflect child ages 3, 5, and 9 years, respectively. The aims of this study included the following: (1) to evaluate the stability of child pro-social skills, receptive vocabulary abilities, and supportive parenting across children ages 3–9 years; and (2) to investigate the transactional nature of concurrent and longitudinal associations of the aforementioned variables by comparing a cross-lagged model, parent effects model, and child-driven model. A parent effects model includes significant pathways from supportive parenting to child pro-social behavior and child receptive language over time, with non-significant pathways from child measures to supportive parenting. In contrast, a child-driven model includes significant paths from child measures to subsequent supportive parenting, and non-significant paths from supportive parenting to later child outcomes. Based on these models, we anticipated a relatively stable increasing pattern of child pro-social skills and child receptive language over time as well as stability in supportive parenting. Further, we expected to observe robust transactional effects for these associations as evidenced by the cross-lagged model better fitting the data in comparison to the parent effects model and the child-driven model. This study extends previous empirical work by examining the concurrent and successive reciprocal associations between child development and supportive parenting within a longitudinal framework, specifically focusing on synergistic child and parent behaviors that can be promoted through intervention. Additionally, the current study advances the current literature base by utilizing data from a large nationally representative sample, measured across multiple child development stages and transitions.

## Methods

### Data Source and Sample

This study used data from the FFCWS, a large population-based birth cohort longitudinal project which followed families from pregnancy through 15 years of age in 20 large United States cities (73) and oversampled children from low-income families and neighborhoods, as well as children born to single mothers from 1998 to 2000. Families were recruited from hospitals and they were interviewed immediately following the focal child's birth (i.e., wave 1, baseline). The present study included families in 18 cities (*n* = 4,374), excluding FFCWS participants in two cities where the pilot study was carried out and thus measurement administration varied initially. The average age of primary caregivers at baseline was 25.29 (SD = 6.05) ranging from 15 to 43 years. The FFCWS followed up with all families when the focal child (boys = 2,276; 52%) was ~1, 3, 5, and 9 years old (i.e., waves 2–5). Participants self-reported mother race (30% White, 51.3% Black or African-American, 2.7% Asian or Pacific Islander, 2.6% American Indian, Eskimo, or Aleut, and 13.5% Other, not specified) and ethnicity (21.7% White, non-Hispanic, 49% Black, non-Hispanic, 25.2% Hispanic, and 3.9% Other). The current study utilized a series of data measures collected through telephone interviews, in-home interviews with a primary caregiver, and in-home observations of family factors as well as child behavior.

### Measures

#### Pro-social Behaviors

Children's pro-social behaviors were assessed using the *Adaptive Social Behavior Inventory* [ASBI; (74)] at ages 3, 4, and 9 years, based on interviews completed over the phone or in-person with primary caregivers. The measure evaluates children's social competence using two subscales of positive social engagement behavior, Express and Comply, based on a 3-point scale (0 = not true to 2 = very or often true). The FFCWS utilized nine items for 3-year study, and 13 items for 5-year and 9-year study after adaptation. In the current study, we created latent variables that consistently reflect children's prosocial behaviors within each wave by applying a cut-off threshold of factor loading below 0.50 to the measurement model and below 0.175 to the correlation matrix including all items, resulting in seven items, 11 items, and 13 items for each wave. Cronbach's αs indicated 0.69, 0.80, and 0.91 at each year, respectively.

#### Receptive Vocabulary

Children's receptive vocabulary was evaluated using standard scores from the *Peabody Picture Vocabulary Test, Third Edition* [PPVT-3; (75)] with children at 3, 5, and 9 years of age. In-home administration procedures included the interviewer reading a word aloud and asking the child to indicate which picture best corresponded to the word from a set of four pictures choices on an easel.

#### Supportive Parenting

Supportive parenting was measured with the *Home Observation for Measurement of the Environment* [HOME; (76)] at 3, 5, and 9 years of age. Trained interviewers assessed supportive parenting behaviors through in-home observations while primary caregivers were interacting with their children. The FFCWS used nine items for wave 3 of the study, eight items for wave 4 of the study, and seven items for wave 5 of the study which were all selected from the original HOME scale. Each item was scored as a binary variable (0 = no or 1 = yes) with higher values indicating more parental responsivity and warmth. Some scale items were included in Waves 3–5 (e.g., parent's voice conveyed positive feelings), however most scale items differed slightly across waves based on child age. For instance, in Wave 3 the observer reported on whether the parent told the name of an object or person and whether the parent kissed or caressed the child, while in Wave 4–5 the observer reported on whether the parent encouraged the child to contribute to conversation and whether the parent helped the child demonstrate an achievement or skill. In this study, we created latent variables that reliably reflect supportive parenting behaviors within each wave by applying a cut-off threshold of factor loadings below 0.50 to the measurement model and below 0.175 to the correlation matrix of all items, resulting in eight items, seven items, and six items for waves 3, 4, and 5, respectfully. Cronbach's αs indicated 0.80, 0.79, and 0.73 at each corresponding wave.

#### Covariates

Given the sample's heterogeneity in risk factors, we additionally controlled for early contextual factors such as child gender and family risk factors measured at baseline (i.e., lack of annual income earned, single parent status, low education, and severe depression) while examining the stability and magnitude of effects observed for these developmental trajectories. A cumulative family risk perspective has been established as an effective strategy for describing the accumulation of numerous risk factors as related to children's maladjustment, rather than presence of a single risk factor (77, 78). Researchers have tried to identify the influence of concurrent, cumulative risk factors by utilizing cumulative risk models wherein a cumulative risk index is calculated by summing each item dichotomized as presence on a value of 1 or absence on a value of 0 (79). Accordingly, the present study considered three variables that may influence observed effects between variables of interest (i.e., child pro-social behavior and receptive vocabulary and supportive parenting). These covarying variables included the following: child gender, mother race, and an index of family risk factors. The family risk index variable was constructed by tallying and summing four indicators (0 = no or 1 = yes) such as no earnings in the past year (70%), single parenthood (41%), <12 years of schooling (33%), and meeting depression criteria (20%) [see (80)].

### Analytical Strategies

The current study conducted a series of Structural Equation Modeling (SEM) tests using Mplus Version 8 (81) with full-information maximum likelihood (FIML) estimation. Specifically, a cross-lagged panel design model was assessed to examine bidirectional effects between child development (i.e., pro-social skills and receptive vocabulary) and supportive parenting. Individual models were evaluated and compared until the best-fitting, most parsimonious model was determined using Chi-square difference tests. The following four criteria were used as model fit indices: (1) the comparative fit index (CFI), (2) the Tucker–Lewis index (TLI), (3) the root mean square error of approximation (RMSEA), and (4) the standardized root mean square residual (SRMR). While CFI and TLI values of over 0.90 and 0.95 suggest a well-specified model, RMSEA and SRMR values close to or lower than 0.08 or indicate acceptable and excellent fit between the model and the dataset (82, 83).

To investigate the primary aims of this study, we estimated and compared a series of individual models while controlling for additional circumstantial factors including child gender, maternal race, and family risk factors evaluated at baseline (i.e., lack of annual income earned, single motherhood, low education, and severe depression) until a parsimonious, best fitting model was identified. Accordingly, we investigated three time points when children were 3, 5, and 9 years old using the following four suggested models: (1) a stability model testing stability of child pro-social behavior, child receptive vocabulary, and supportive parenting, (2) a cross-lagged model testing concurrent and longitudinal associations of child pro-social behavior, child receptive vocabulary, and supportive parenting behaviors over time, (3) a parent-effects model testing how supportive parenting relates to later child pro-social behavior and receptive vocabulary, and (4) a child-driven model testing how child pro-social behavior and receptive vocabulary relate to later supportive parenting behaviors.

#### Missing Data

The FIML approach is a useful missing data estimation method which calculates unbiased parameter estimates and standard errors. This method provides parameter estimates while maximizing the likelihood of the underlying distributions of incomplete data with missingness so that all the information available is fully used (84). Group comparison tests were conducted to see if there were differences in demographics between the original sample from 18 cities (*n* = 4,374) and the remaining sample in the FFCWS project until Wave 5 (*n* = 2,925). Family information, including child gender, maternal age, maternal race, maternal education, and family income measured at baseline, was examined using independent samples *t*-tests. There were no significant differences in child gender, *t*_(7, 296)_ = 0.44, *p* = 0.66, mother's age, *t*_(7, 292)_ = 1.44, *p* = 0.15, and maternal race, *t*_(7, 170)_ = −0.93, *p* = 0.35, between the two groups. There were differences in maternal education and family income such that the remaining sample was more likely to have higher education, *t*_(7, 290)_ = 3.38, *p* = 0.001, and greater household income, *t*_(7, 296)_ = 3.86, *p* < 0.001, compared to the original sample. Low maternal education level and poverty were included as covariates for all models tested in the current study.

## Results

### Stability and Cross-Lagged Models

Simultaneous and prospective associations among child pro-social behaviors, receptive vocabulary, and supportive parenting at child ages 3, 5, and 9 years, were evaluated with stability and cross-lagged models, then compared to each other using chi-square difference tests. Descriptive statistics and effect sizes for all associations across constructs are shown in Table 1. The model fit indices indicated CFI = 0.89, TLI = 0.88, RMSEA = 0.03, SRMR = 0.04 for the stability and cross-lagged models. Comparison of the stability and cross-lagged models yielded a change in χ^2^ of 77.30 (*p* < 0.001) with a difference of 4 degrees of freedom such that the additional free parameters in the cross-lagged model provided a better fit than the stability model (see Table 2). Therefore, the cross-lagged model was selected as the better fitting model. Favoring the cross-lagged model to the stability model suggested that the three constructs in the proposed model influenced each other over time.

**Table 1:** Descriptive statistics and Pearson correlations among study variables.

**Variables**	**1**	**2**	**3**	**4**	**5**	**6**	**7**	**8**	**9**
1. PSB3	1.00								
2. PSB4	0.33^***^	1.00							
3. PSB5	0.291^***^	0.313^***^	1.00						
4. RV3	0.195^***^	0.199^***^	0.168^***^	1.00					
5. RV4	0.178^***^	0.232^***^	0.227^***^	0.504^***^	1.00				
6. RV5	0.195^***^	0.185^***^	0.214^***^	0.465^***^	0.624^***^	1.00			
7. SP3	0.196^***^	0.171^***^	0.139^***^	0.280^***^	0.208^***^	0.202^***^	1.00		
8. SP4	0.091^***^	0.211^***^	0.119^***^	0.157^***^	0.277^***^	0.232^***^	0.190^***^	1.00	
9. SP5	0.014	0.066^**^	0.078^***^	0.049^*^	0.109^***^	0.098^***^	0.101^***^	0.125^***^	1.00
	PSB3	PSB4	PSB5	RV3	RV4	RV5	SP3	SP4	SP5
*N*	2,771	2,828	2,866	2,182	2,235	3,014	1,826	1,987	2,698
*M*	1.69	1.73	1.47	85.52	92.99	92.87	0.87	0.75	0.74
*SD*	0.32	0.29	0.44	16.69	15.96	14.68	0.21	0.27	0.27
Range	2.00	2.00	2.00	97	99	115	1.00		
Skew	−1.23	−1.52	−0.98	−0.39	−0.55	0.43	−2.08	−0.93	−0.95

*PSB, child pro-social behavior; RV, child receptive vocabulary; SP, supportive parenting, labels of 3, 4, or 5 indicates Fragile Families data wave 3, 4, or 5 corresponding to child age 3, 5, or 9 years, respectively*.

* *p < 0.05,*

** *p < 0.01,*

*** *p < 0.001*.

**Table 2:** Chi square difference test across nested models of child prosocial behavior, receptive language, and supportive parenting.

**Model**	***x*^2^-test**				**Change in *x*^2^-test**		
	***x* ^2^**	**df**	***x*^2^/df**	***p***	***x* ^2^**	**df**	***p***
1. Stability	5,981.05	1,556	3.84	<0.001			
2. Cross-lagged	5,903.76	1,552	3.80	<0.001			
3. Stability vs. cross-lagged					77.30	4	<0.001
4. Parent effects	5,897.45	1,548	3.81	<0.001			
5. Cross-lagged vs. parent effects					6.31	4	0.178
6. Child-driven	5,871.89	1,544	3.8	<0.001			
7. Cross-lagged vs. child-driven					31.87	8	<0.001
8. Child-driven (trimmed)	5,880.65	1,550	3.8	<0.001			

*N = 3,382. Model fit indices for all models include CFI = 0.89, TLI = 0.88, RMSEA = 0.03, SRMR = 0.04*.

In the cross-lagged model, child pro-social behavior at age 3 and 5 years, β = 0.41, *p* < 0.001, and at 5 and 9 years, β = 0.33, *p* < 0.001, was positively correlated. Child receptive vocabulary at age 3 and 5 years, β = 0.47, *p* < 0.001, and that of children age 5 and 9 years, β = 0.61, *p* < 0.001, was also positively correlated. Supportive parenting at child age 3 and 5 years, β = 0.20, *p* < 0.001, and that of child 5 and 9 years, β = 0.18, *p* < 0.001, was positively correlated. At child age 3 years, pro-social behavior was positively related to receptive vocabulary, β = 0.22, *p* < 0.001, and to supportive parenting, β = 0.30, *p* < 0.001, and receptive vocabulary was positively associated with supportive parenting, β = 0.24, *p* < 0.001. At child age 5 years, pro-social behavior was positively related to receptive vocabulary, β = 0.10, *p* < 0.001, and to supportive parenting, β = 0.21, *p* < 0.001, and receptive vocabulary was positively associated with supportive parenting, β = 0.25, *p* < 0.001. At age 9 years, pro-social behavior was positively related to receptive vocabulary, β = 0.06, *p* = 0.013, and to supportive parenting, β = 0.11, *p* < 0.001, and receptive vocabulary was positively associated with supportive parenting, β =0.06, *p* = 0.012. Pro-social behavior of children at 3 years of age was positively linked to receptive vocabulary of 5 years, β = 0.11, *p* < 0.001, pro-social behavior at 5 years of age, and receptive vocabulary at age 9 years, β = 0.05, *p* = 0.007. Receptive vocabulary of 3-year-old children was positively linked to pro-social behavior of children at age 5 years, β = 0.13, *p* < 0.001, and receptive vocabulary at age 5 years was associated with pro-social behavior at age 9-years, β = 0.06, *p* = 0.012.

### Parent Effects Model

Next, a parent effects model was tested to assess how supportive parenting behaviors are related to child pro-social behavior and receptive vocabulary over time, while taking into account the stability of each factor. The model fit indices of the parent effects model were CFI = 0.89, TLI = 0.88, RMSEA = 0.03, SRMR = 0.04. Comparison of the cross-lagged and parent effects models indicated that the cross-lagged model was a better fitting model, Δχ^2^(4) = 6.31(n.s.) such that supportive parenting behaviors were not significantly altering the patterns of later child pro-social behavior or receptive vocabulary. Rather, the model including associations between supportive parenting behaviors and child outcomes over time more precisely described observed patterns of change between *all* of the individual data points in the sample. The parent effects model, which omitted paths signifying correlations between child outcomes and subsequent parenting behaviors, did not yield a more accurate depiction of the observed associations between these constructs.

### Child-Driven Model

**Figure 1:**
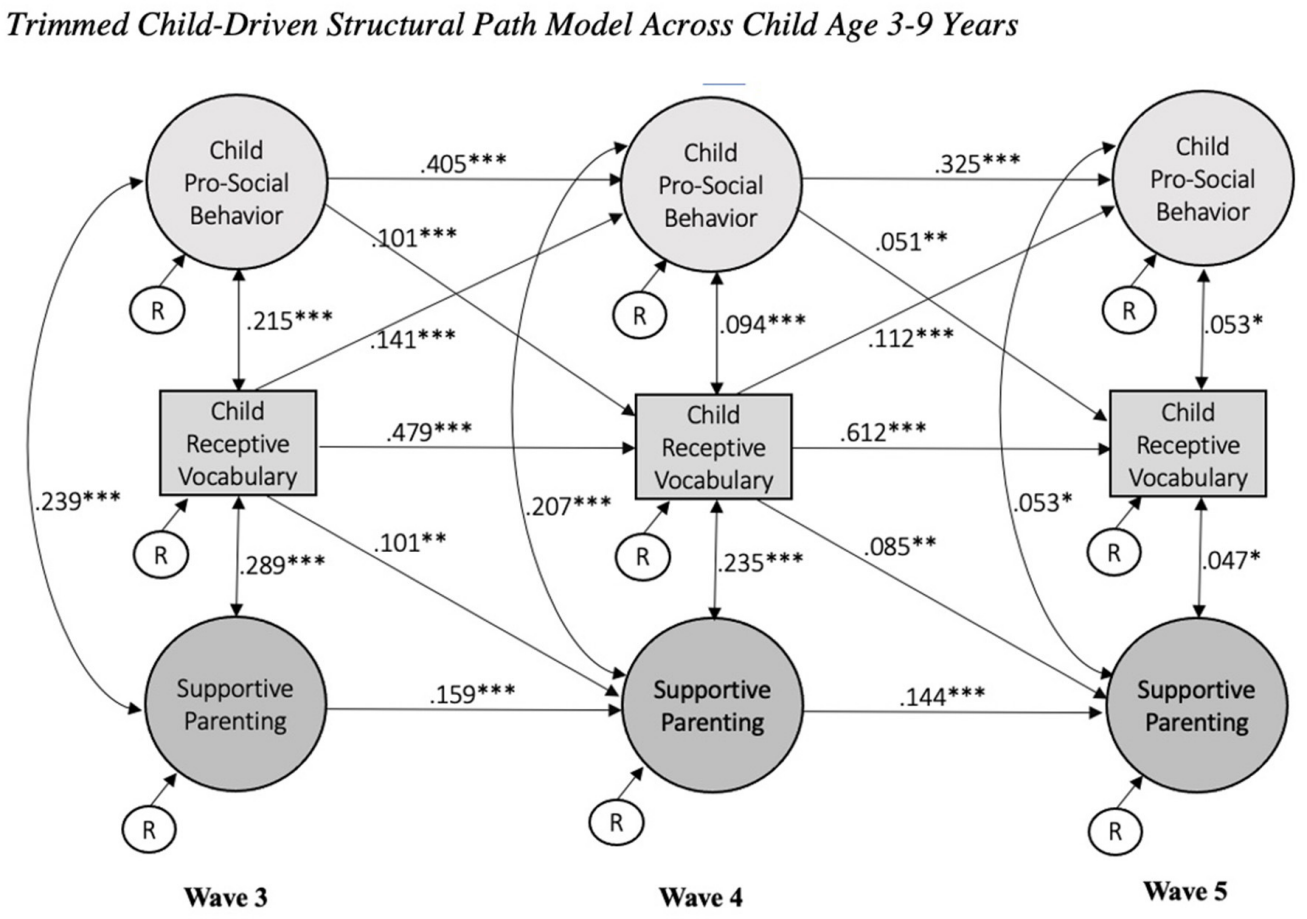
Trimmed child-driven structural path model across child age 3–9 years. Focal child ages are 3, 5, and 9 years at wave 3, 4, and 5, respectively. Model controlled for child gender, mother's race, and family risk at baseline. Stability and cross-lagged models include only child pro-social behavior and receptive vocabulary. Standardized coefficients presented. *p < 0.05, **p < 0.01, ***p < 0.001.

A child-driven model was tested to identify whether patterns of child pro-social behavior and receptive vocabulary related to later supportive parenting, while taking into account the stability of each factor. The model fit indices in the child-driven model were CFI = 0.89, TLI = 0.88, RMSEA = 0.03, SRMR = 0.04. Results of comparing the cross-lagged and child-driven models showed a change in χ^2^ of 31.87 (*p* < 0.001) with a difference of 8 degrees of freedom, such that the additional free parameters in the child-driven model provided a better fit than the cross-lagged model. Favoring the child-driven model to the cross-lagged indicated that early child development, including pro-social behavior and receptive vocabulary, was related to later supportive parenting. The child-driven model was selected as the most parsimonious, better fitting model and was then trimmed based on significance levels (*p* < 0.05) of standardized parameter estimates of direct effects. The trimmed child-driven model did not show a significant decline in the model fit value, Δχ^2^ (6) = 8.76 (n.s.), and was selected as the best model with model fit indices, CFI = 0.89, TLI = 0.88, RMSEA = 0.03, SRMR = 0.04. Figure 1 shows standardized path coefficients; the significant standardized coefficients ranged from 0.047 to 0.612, *p*-values ranged from < 0.000 to 0.047. The 95% lower and upper limits of bias-corrected bootstrap confidence intervals (95% BC CIs) did not include zero; for example, 95% CI [0.574, 0.653] for β = 0.612 and 95% CI [0.003, 0.091] for β = 0.053.

### Models by Maternal Race and Child Gender

To identify potential conditional associations in the child-driven model, maternal race and child gender were further evaluated as grouping variables. When maternal race was incorporated as a grouping variable with child gender and family risk as control variables, the model failed to converge. In the final child-driven model selected, maternal race was only significant in relation to child prosocial behavior at age 5 years and supportive parenting at child age 9 years (β = −0.07, *p* < 0.001). Inclusion of maternal race in all models did not yield meaningful differences in model fit or parameter estimations. Therefore, race was not found to be an influential factor in our study.

**Figure 2:**
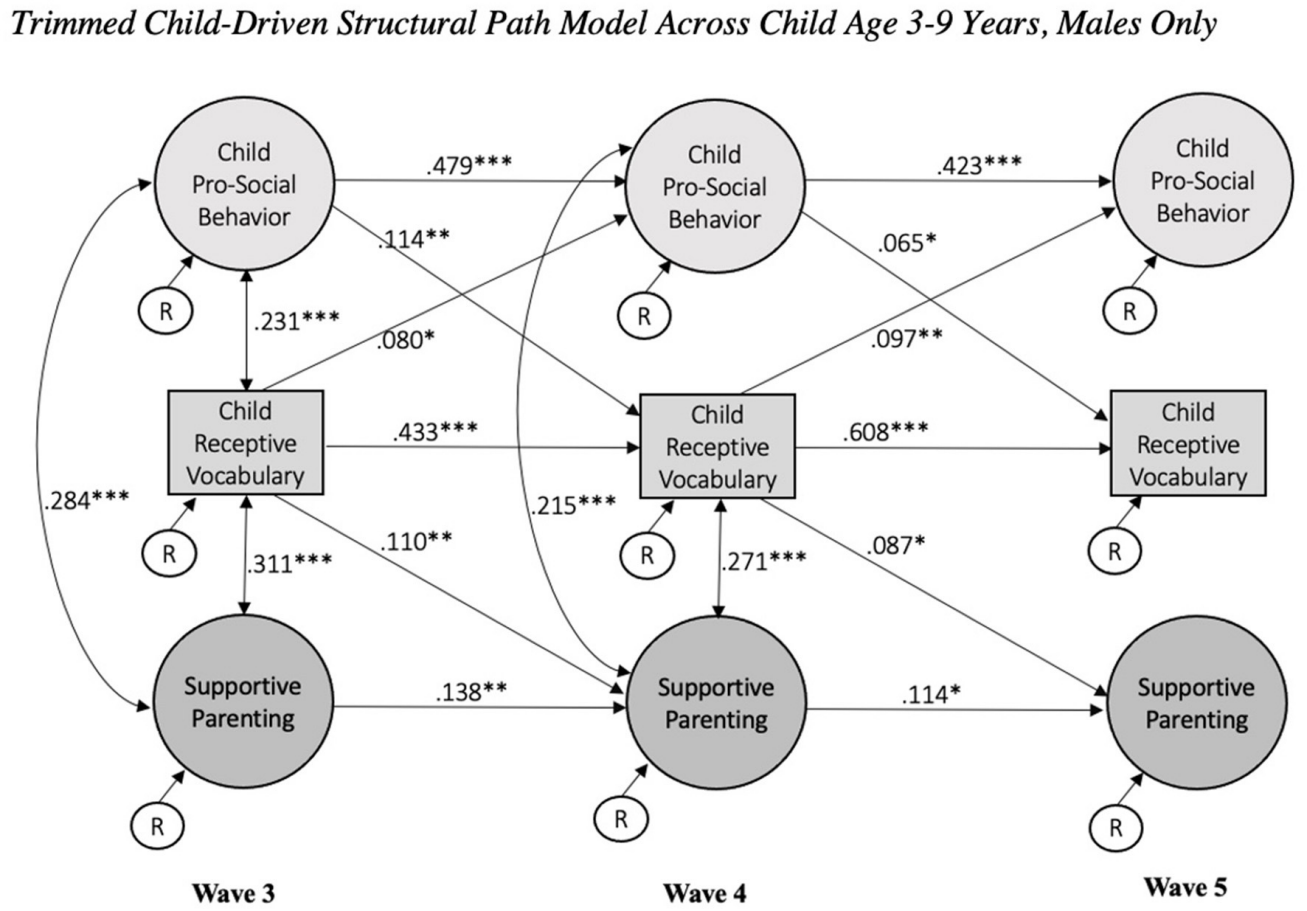
Trimmed child-driven structural path model across child age 3–9 years, males only. Focal child ages are 3, 5, and 9 years at wave 3, 4, and 5, respectively. Model controlled for child gender, mother's race, and family risk at baseline. Standardized coefficients presented. *p < 0.05, **p < 0.01, ***p < 0.001.

**Figure 3:**
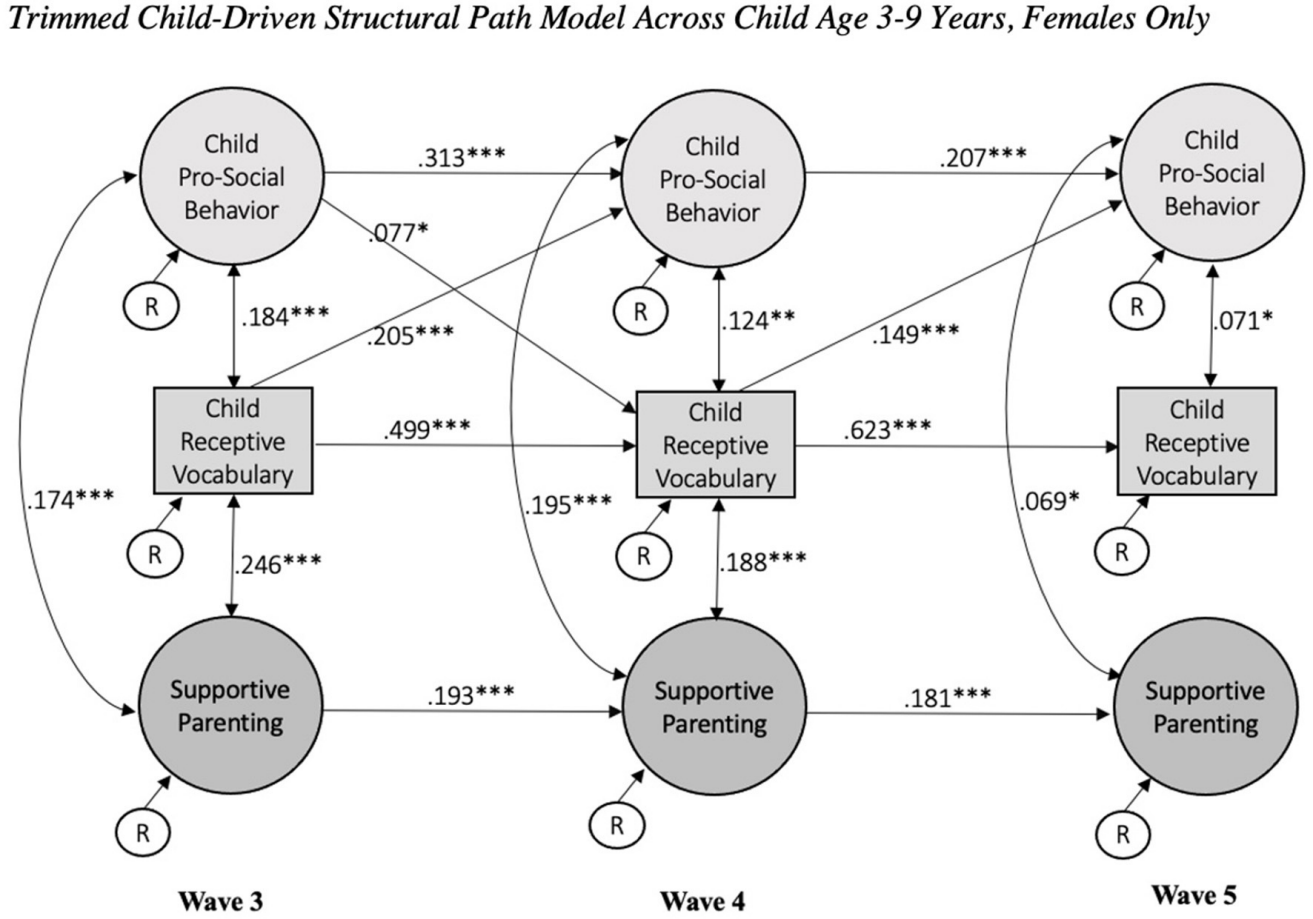
Trimmed child-driven structural path model across child age 3–9 years, females only. Focal child ages are 3, 5, and 9 years at wave 3, 4, and 5, respectively. Model controlled for child gender, mother's race, and family risk at baseline. Standardized coefficients presented. *p < 0.05, **p < 0.01, ***p < 0.001.

Then, a model with child gender as a grouping variable was estimated to probe gender differences and gauge next steps for multi-group investigations. In contrast to our non-significant findings for race, child gender contributed to differences in parameter estimation and model fit, χ^2^(3, 100) = 7,833.89 (*p* < 0.001), CFI = 0.88, TLI = 0.87, RMSEA = 0.03, SRMR = 0.04. In the final child-driven model selected, child gender was significant in relation to child prosocial behavior at all waves (in increasing wave order: β = 0.06, *p* = 0.01; β = 0.05, *p* = 0.03; β = 0.04, *p* = 0.04), child receptive language at all waves (β = 0.05, *p* = 0.01; β = 0.04, *p* = 0.03; *p* = −0.08; β = 0.04, *p* < 0.001), and supportive parenting only at child age 5 years (β = 0.06, *p* = 0.02). See Figures 2, 3 for standardized parameter estimations specific to males and females, respectively. The 95% CIs did not include zero; for instance, 95% CI [0.537, 0.661] for β = 0.608 and 95% CI [0.002, 0.176] for β = 0.087 in the males only child-driven model, and 95% CI [0.580, 0.665] for β = 0.623 and 95% CI [0.010, 0.147] for β = 0.077 in the females only child-driven model.

In the males only child-driven model, the concurrent correlations in association between child pro-social behavior and receptive vocabulary were non-significant at age 5 and 9 years. Additionally, the association between males' pro-social behavior and supportive parenting at age 9 years was also non-significant. In the females only model, child-driven effects observed were limited to child pro-social behavior; that is, receptive vocabulary at child age 3 and 5 years was not significantly associated with supportive parenting at child age 5 and 9 years, respectively. The concurrent association between females' pro-social behavior at age 5 years and receptive vocabulary at age 9 years was also non-significant. With incorporation of the grouping variable, both males and females only models no longer indicated a significant relation between receptive child vocabulary and supportive parenting at age 9 years.

## Discussion

This study provides novel evidence and insight regarding early parent-child dynamics and associated patterns in child development for children from at-risk, or fragile, families. Specifically, these findings contribute support for a child-driven model to best explain the reciprocal associations between child pro-social skills, child receptive language, and supportive parenting. In the final model, autoregressive paths for both child development dimensions of interest, receptive language and pro-social skills, remained significant across all child ages measured. The autoregressive paths for supportive parenting also remained significant over time. Associations between child development measures and later supportive parenting behaviors were consistently found to be significant across waves, however the associations between supportive parenting and later child development measures were non-significant, hence confirmation of the proposed child-driven effects model. Upon evaluation of differences in estimated model parameters and significant paths attributed to child gender, the relation between concurrent pro-social behavior and receptive vocabulary was non-significant for boys at age 5 and 9 years. And, group differences indicated girls' receptive vocabulary skills, unlike pro-social behavior, were not associated with changes in supportive parenting over time. This investigation builds upon prior studies that have investigated child development outcomes utilizing a dynamic systems approach (1, 34, 85), emphasizing that child skill development in various domains occurs as a result of interactions between internal systems (e.g., child language skills) and external systems (e.g., supportive parenting). By testing a series of SEM models with a large longitudinal dataset from fragile families, our results provide additional compelling support for feedback loops and phase transitions as evidenced by the cross-lagged associations and shifts in association magnitude over time (86, 87). Further, by focusing on a sample of interest based on familial risk this investigation draws targeted attention to intervention approach considerations warranted including timeliness of parent-driven vs. child-driven parent outcomes.

When comparing within- and across-time models of relations between child pro-social behavior, child receptive language, supportive parenting with and without cross-lagged effects accounted for, we confirmed change in both child and parent factors of interest over time shifted collectively as modeled in the cross-lagged model rather than independently as modeled in the stability model. That is, social skill development and early child language do not occur in a continuous vacuum, and early parenting behaviors fluctuate as well. Patterns of correlation for child pro-social behavior were stable over time, with the strongest change in association magnitude observed in the transition from age 3 to 5 years. This is consistent with prior empirical evidence emphasizing the pre-school to primary school transition period as a crucial window of time for honing early social skills (31, 88). Similarly, patterns of correlation for child receptive vocabulary also remained stable over time, although the strongest association was observed for the transition for children from age 5 to 9 years. Again, this finding is corroborated by prior research identifying the early to middle childhood period as a distinct phase of receptive vocabulary development and important period for intervention (89, 90). As expected, the magnitude of association between child pro-social behavior and child receptive vocabulary remained relatively consistent over time.

While we anticipated the reciprocal parent-child factor associations, subsequent model comparisons indicated that not only did supportive parenting behaviors fluctuate with these child factors—supportive parenting fluctuated based on association with the child factors as evidenced by the child-driven model better fitting the data than the transactional model and the parent-driven effects model. This finding suggests that targeting early child developmental skills such as social behavior and language skills in a variety of learning environments (e.g., home, preschool or daycare, and primary school) has the potential to elicit positive, indirect effects on supportive parenting over time. Notably, prior research has identified attentional and emotional regulation as a mechanism of change between early supportive parenting qualities (e.g., warmth, lack of hostility) and later child pro-social behavior (53). Consistent with Healy et al. (54), we assert that parent-child engagement quality and depth of communication may be critical contributing factors for child pro-social skills, above and beyond supportive parenting qualities. We propose that our findings for child-driven effects can be explained by parents reinforcing child early social engagement strengths with facilitative parenting, thus parents and children engage in more frequent and complex social routines when children are effectively regulating their social attention and understanding parent communication. Our results for gender differences were unexpected, however we suggest these findings can be explained by girls' strengths in early pro-social skills compared to boys (91) exacerbating the child-driven effects observed for social skills over time rather than receptive language. Contrary to the findings from Slot et al. (92), the current study found language to be significantly associated with of both boys' and girls' social skills over time. Since that study's measure of social skills was more holistic in comparison to the current study's more precise evaluation of pro-social behavior, we attribute our lack of corroboration with Slot et al. (92) may be due to underlying gender differences in aspects of social-emotional skills including self-regulation and activity level (93, 94).

Given the sample of interest in this study and the added environmental, financial, and emotional stress often experienced by these families, prioritizing child targets can not only bolster internal child outcomes but also elicit buffering effects for the entire family system. Further, the magnitude of concurrent association between supportive parenting and both child pro-social behavior and child receptive vocabulary was observed to be strongest when children are 3 years of age, and concurrent, as well as cross-lagged, correlations declined in strength over time when associations were adjusted for prior achievement status or behavior. That is, these results highlight the necessity of intervention and prevention practices for at-risk children and families as early as possible to optimize these reciprocal and child-driven effects. The loss of significant pathways between concurrent measures of interest at 9 years of age for boys in the current study also suggests that the transactional effects of positive parent-son social engagement routines may be more instrumental for child development outcomes when targeted before beginning primary school. Since parent-driven effects were not observed over time for supportive parenting behaviors specifically including warm and verbally responsive behavior, we also emphasize the need for targeted interventions to include parent coaching on communication quality such as aligning timely verbal responses to child interests in a distraction free environment. Thus, optimal parent-child interaction routines may utilize previously established mechanisms of growth, such as child emotion and attention regulation, through engaging play activities in addition to conversational dialogue.

### Limitations

The present study includes several strengths including utilization of advanced statistical analysis, SEM, with a large longitudinal dataset and a variety of observational as well as parent-reported measures. However, we acknowledge some limitations and provide relevant recommendations for future studies. While the authors selected a cross-lagged panel model based on the goal of evaluating collective patterns over time rather than within-person dynamics, we acknowledge this type of model assumes that model effects are fixed across individuals. It is possible that measurement of between-person variance with a random-intercept model could yield different association patterns. We acknowledge potential unobserved confounds that were not adjusted for, resulting in a weak set of controls. More specifically: while we were able to account for shared measurement variance by including latent factors for child pro-social behavior and supportive parenting, receptive language was incorporated in all models with a single indicator factor based on the language variables measured in the FFCWS. This analysis could have been strengthened with incorporation of additional language measures including parent-report and teacher-report measures. Further, future studies could integrate a more comprehensive assessment of language skills by incorporating expressive language measures, analysis of spontaneous speech samples, and early non-verbal communication assessments (e.g., gesture profiles). The current study focused on receptive language, and not expressive language, based on the standardized measures administered in the FFCWS study at these time points. Additionally, future works utilizing SEM could strive to incorporate latent factors with more rigorous consistency across time points.

While all observed variables for pro-social behavior and supportive parenting in this study were obtained from the same measures at each wave, the ASBI (74) and HOME (76), respectively, and each latent factor was evaluated and modified to fulfill inter-item reliability stipulations, item phrasing and quantity of items administered varies across waves based on child norms expected for various developmental periods. Ruling out potentially confounding item variance introduced by item variability between waves may improve validity and subsequent causality assumptions. Given the nature of some of the HOME items (e.g., reporting of observed physical and verbal reprimand, noting access to learning enrichment materials) observed and the sample of interest, we acknowledge inherent limits to measurement with this tool and recommend caution in both analysis and generalization of results in future works with the HOME scale. Further, while the current study evaluated variability in reciprocal effects specific to child gender and maternal race, we urge future work to utilize a more socioeconomically diverse sample and complete a dynamic examination of reciprocal effects in the context of income disparities. Relatedly, although the effect sizes when child gender was used as a grouping variable cannot be directly compared between the two groups, it was obvious that boys and girls showed differences in associations between child development and supportive parenting over time. Future studies utilizing a multi-group approach of comparing effect sizes may advance our understanding of specific differences between genders. Based on data attrition and item consistency over time, the current study utilized an additive risk approach and controlled for family risk factors as a time-invariant variable. We suggest that future studies include careful consideration of both time-invariant and time variant demographic variables and family risk items in future work. Lastly, we acknowledge that the current study strived to evaluate supportive parenting behaviors, but our analysis primarily includes data reflecting mothers' parenting behavior based on the FFCWS oversampling children born to unmarried mothers. Evaluating mothers' and fathers' unique contributions [see (95)] to the reciprocal relations of interest in the current study is an area of need for future research.

## Conclusion

This investigation provides a significant contribution to the current literature examining transactional associations across child development by supporting significant child-driven effects, explaining children have the capability to influence mothers' parenting behaviors. Positive associations between child pro-social behavior and receptive vocabulary, as well as supportive parenting, were observed across early child development. The magnitude of these associations steadily declined over time, highlighting the importance of early synergistic parent-child interactions. Patterns of change in child pro-social behavior skills and supportive parenting remained positive and relatively stable, while observed growth in child receptive vocabulary skills increased in magnitude over time. In summary, by utilizing a large longitudinal sample of at-risk families we have extended prior understanding of transactional patterns in parent-child responsivity and specified how the magnitude and stability of these reciprocal behaviors are dynamically evolving due to child-driven effects across early to middle childhood. Further, we have specified how child pro-social behaviors and receptive vocabulary is concurrently and successively shaped in the context of parent and child responsivity and provided recommendations for early intervention timing and dosage.

## Data Availability Statement

Publicly available datasets were analyzed in this study. This data can be found at: Fragile Families and Child Wellbeing Study, https://opr.princeton.edu/archive/.

## Ethics Statement

This study was exempt from review by the Texas Tech University Institutional Review Board due to the study utilizing completely de-identified secondary data. Written informed consent to participate in this study was provided by the participants' legal guardian/next of kin.

## Author Contributions

JB, SP, MC, and AM designed the study. JB and MC prepared the dataset. SP completed initial analyses and additional analyses. JB developed a first draft of the comprehensive manuscript under AM's guidance and revised manuscript to reflect updated findings. All authors provided feedback and implemented manuscript revisions. All authors contributed to the article and approved the submitted version.

## Funding

The data utilized in this investigation from the Fragile Families and Child Wellbeing Study was supported by R01HD036916, R01HD039135, and R01HD040421, as well as The Annie E. Casey Foundation and the Bill and Melinda Gates Foundation. The funders were not involved in the study design, collection, analysis, interpretation of data, the writing of this article or the decision to submit it for publication.

## Conflict of Interest

The authors declare that the research was conducted in the absence of any commercial or financial relationships that could be construed as a potential conflict of interest.

## Publisher's Note

All claims expressed in this article are solely those of the authors and do not necessarily represent those of their affiliated organizations, or those of the publisher, the editors and the reviewers. Any product that may be evaluated in this article, or claim that may be made by its manufacturer, is not guaranteed or endorsed by the publisher.

## References

[1] Barnett MA Gustafsson H Deng M Mills-Koonce WR Cox M. Bidirectional associations among sensitive parenting, language development, and social competence. Infant Child Dev. (2012) 21:374–93. 10.1002/icd.1750 25126021 PMC4128493

[2] Bornstein MH Hahn CS Putnick DL. Long-term stability of core language skill in children with contrasting language skills. Dev Psychol. (2016) 52:704–16. 10.1037/dev0000111 26998572 PMC4844756

[3] Fadda R Lucarelli L. Mother-infant and extra-dyadic interactions with a new social partner: developmental trajectories of early social abilities during play. Front Psychol. (2017) 8:436. 10.3389/fpsyg.2017.00436 28443038 PMC5387069

[4] Paavola-Ruotsalainen L Lehtosaari J Palomäki J Tervo I. Maternal verbal responsiveness and directiveness: consistency, stability, and relations to child early linguistic development. J Child Lang. (2018) 45:319–39. 10.1017/S030500091700023X 28637528

[5] DeVeney S Cress CJ Lambert M. Parental directiveness and responsivity toward young children with complex communication needs. Int J Speech Lang Pathol. (2016) 18:53–64. 10.3109/17549507.2015.1081282 28425365

[6] Helland SS Roysamb E Wang MV Gustavson K. Language difficulties and internalizing problems: bidirectional associations from 18 months to 8 years among boys and girls. Dev Psychopathol. (2018) 30:1239–52. 10.1017/S0954579417001559 29117871

[7] Abaied JL Stanger SB. Socialization of coping in a predominantly female sample of caregivers: contributions to children's social adjustment in middle childhood. J Fam Psychol. (2017) 31:958–64. 10.1037/fam0000342 29083207

[8] Rodkin PC Ryan AM Jamison R Wilson T. Social goals, social behavior, and social status in middle childhood. Dev Psychol. (2013) 49:1139–50. 10.1037/a0029389 22822934

[9] Danzig AP Dyson MW Olino TM Laptook RS Klein DN. Positive parenting interacts with child temperament and negative parenting to predict children's socially appropriate behavior. J Soc Clin Psychol. (2015) 34:411–35. 10.1521/jscp.2015.34.5.411 28824223 PMC5560516

[10] Feldman R. Mutual influences between child emotion regulation and parent–child reciprocity support development across the first 10 years of life: Implications for developmental psychopathology. Dev Psychopathol. (2015) 27:1007–23. 10.1017/S0954579415000656 26439059

[11] Liu P Kryski KR Smith HJ Joanisse MF Hayden EP. Transactional relations between early child temperament, structured parenting, and child outcomes: a three-wave longitudinal study. Dev Psychopathol. (2019) 32:1–11. 10.1017/S0954579419000841 31298177

[12] Sameroff A. The transactional model of development: how children and contexts shape each other. Am Psychol Assoc. (2009). 10.1037/11877-00029170935

[13] Blair C Raver CC. School readiness and self-regulation: a developmental psychobiological approach. Annu Rev Psychol. (2015) 66:711–31. 10.1146/annurev-psych-010814-015221 25148852 PMC4682347

[14] Jones DE Greenberg MT Crowley DM. Early social-emotional functioning and public health: the relationship between kindergarten social competence and future wellness. Am J Public Health. (2015) 105:2283–90. 10.2105/AJPH.2015.302630 26180975 PMC4605168

[15] Letourneau NL Duffett-Leger L Levac L Watson B Young-Morris C. Socioeconomic status and child development: a meta-analysis. J Emot Behav Disord. (2013) 21:211–24. 10.1177/1063426611421007

[16] Halfon N Larson K Son J Lu M Bethell C. Income inequality and the differential effect of adverse childhood experiences in US children. Acad Pediatr. (2017) 17:S70–8. 10.1016/j.acap.2016.11.007 28865663

[17] Schenck-Fontaine A Panico L. Many kinds of poverty: three dimensions of economic hardship, their combinations, and children's behavior. Demography. (2019) 56:2279–305. 10.1007/s13524-019-00833-y 31808103 PMC7172985

[18] Conger RD Wallace LE Sun Y Simons RL McLoyd VC Brody GH. Economic pressure in African American families: a replication and extension of the family stress model. Dev Psychol. (2002) 38:179–93. 10.1037/0012-1649.38.2.179 11881755

[19] Romeo RR Leonard JA Robinson ST West MR Mackey AP Rowe ML. Beyond the 30-million-word-gap: children's conversational exposure is associated with language-related brain function. Psychol Sci. (2018) 29:700–10. 10.1177/0956797617742725 29442613 PMC5945324

[20] Smith RL Stagnitti K Lewis AJ Pépin G. The views of parents who experience intergenerational poverty on parenting and play: a qualitative analysis. Child. (2015) 41:873–81. 10.1111/cch.12268 26119480

[21] Barnett MA Paschall KW Mastergeorge AM Cutshaw CA Warren SM. Influences of parent engagement in early childhood education centers and the home on kindergarten school readiness. Early Child Res Q. (2020) 53:260–73. 10.1016/j.ecresq.2020.05.005

[22] Flouri E Midouhas E Joshi H Tzavidis N. Emotional and behavioural resilience to multiple risk exposure in early life: the role of parenting. Eur Child Adolesc Psychiatry. (2014) 24:745–55. 10.1007/s00787-014-0619-7 25300919

[23] Jeon HJ Peterson CA DeCoster J. Parent-child interaction, task-oriented regulation, and cognitive development in toddlers facing developmental risks. J Appl Dev Psychol. (2013) 34:257–67. 10.1016/j.appdev.2013.08.002

[24] Carlson MT Sonderegger M Bane M. How children explore the phonological network in child-directed speech: a survival analysis of children's first word productions. J Mem Lang. (2014) 75:159–80. 10.1016/j.jml.2014.05.005 25089073 PMC4115338

[25] Liszkowski U. Two sources of meaning in infant communication: preceding action contexts and act-accompanying characteristics. Philos Trans. (2014) 369:1–9. 10.1098/rstb.2013.0294 25092662 PMC4123673

[26] Chládková K Paillereau N. The what and when of universal perception: a review of early speech sound acquisition. Lang Learn. (2020) 70:1136–82. 10.1111/lang.12422

[27] Tendera A Rispoli M Senthilselvan A Loucks TM. Early speech rate development: a longitudinal study. J Speech Lang Hear Res. (2019) 62:4370–81. 10.1044/2019_JSLHR-19-00145 31830834

[28] Kemple KM Lee I Ellis SM. The impact of a primary prevention program on preschool children's social–emotional competence. Early Childh Educ J. (2019) 47:641–52. 10.1007/s10643-019-00963-3

[29] Warneken F Tomasello M. Varieties of altruism in children and chimpanzees. Trends Cogn Sci. (2009) 13:397–402. 10.1016/j.tics.2009.06.008 19716750

[30] Cochet H Byrne RW. Communication in the second and third year of life: relationships between nonverbal social skills and language. Infant Behav Dev. (2016) 44:189–98. 10.1016/j.infbeh.2016.07.003 27450099

[31] Hukkelberg S Keles S Ogden T Hammerstrøm K. The relation between behavioral problems and social competence: a correlational meta-analysis. BMC Psychiatry. (2019) 19:1–14. 10.1186/s12888-019-2343-9 31706279 PMC6842530

[32] McKean C Law J Mensah F Cini E Eadie P Frazer K. Predicting meaningful differences in school-entry language skills from child and family factors measured at 12 months of age. Int J Early Childh. (2016) 48:329–51. 10.1007/s13158-016-0174-0

[33] Doyle C Cicchetti D. From the cradle to the grave: the effect of adverse caregiving environments on attachment and relationships throughout the lifespan. Clin Psychol. (2017) 24:203–17. 10.1111/cpsp.12192 28924334 PMC5600283

[34] Ayoub C Vallotton CD Mastergeorge AM. Developmental pathways to integrated social skills: the roles of parenting and early intervention. Child Dev. (2011) 82:583–600. 10.1111/j.1467-8624.2010.01549.x 21410921

[35] Bater LR Jordan SS. Child routines and self-regulation serially mediate parenting practices and externalizing problems in preschool children. Child Youth Care Forum. (2016) 46:243–59. 10.1007/s10566-016-9377-7

[36] Law J Rush R McBean K. The relative roles played by structural and pragmatic language skills in relation to behaviour in a population of primary school children from socially disadvantaged backgrounds. Emot Behav Difficult. (2013) 19:28–40. 10.1080/13632752.2013.854960

[37] Raby KL Roisman GI Fraley RC Simpson JA. The enduring predictive significance of early maternal sensitivity: social and academic competence through age 32 years. Child Dev. (2015) 86:695–708. 10.1111/cdev.12325 25521785 PMC4428971

[38] Spencer S Clegg J Stackhouse J Rush R. Contribution of spoken language and socio-economic background to adolescents' educational achievement at age 16 years. Int J Lang Commun Disord. (2017) 52:184–96. 10.1111/1460-6984.12264 27432281

[39] Weisleder A Fernald A. Talking to children matters: early language experience strengthens processing and builds vocabulary. Psychol Sci. (2013) 24:2143–52. 10.1177/0956797613488145 24022649 PMC5510534

[40] Bratsch-Hines ME Carr R Zgourou E Vernon-Feagans L Willoughby M. Infant and toddler child-care quality and stability in relation to proximal and distal academic and social outcomes. Child Dev. (2020) 91:1854–64. 10.1111/cdev.13389 32662886 PMC7793551

[41] Conway LJ Levickis PA Smith J Mensah F Wake M Reilly S. Maternal communicative behaviours and interaction quality as predictors of language development: findings from a community-based study of slow-to-talk toddlers. Int J Lang Commun Disord. (2018) 53:339–54. 10.1111/1460-6984.12352 29218767

[42] Nozadi SS Spinrad TL Eisenberg N Bolnick R Eggum-Wilkens ND Smith CL. Prediction of toddlers' expressive language from maternal sensitivity and toddlers' anger expressions: a developmental perspective. Infant Behav Dev. (2013) 36:650–61. 10.1016/j.infbeh.2013.06.002 23911594 PMC3858491

[43] Altschul I Lee SJ Gershoff ET. Hugs, not hits: warmth and spanking as predictors of child social competence. J Marriage Family. (2016) 78:695–714. 10.1111/jomf.12306 34584295 PMC8475779

[44] Girard LC Doyle O Tremblay RE. Maternal warmth and toddler development: support for transactional models in disadvantaged families. Eur Child Adolesc Psychiatry. (2017) 26:497–507. 10.1007/s00787-016-0913-7 27771763

[45] Vallotton CD Harewood T Ayoub CA Pan B Mastergeorge AM Brophy-Herb H. Buffering boys and boosting girls: the protective and promotive effects of Early Head Start for Children's expressive language in the context of parenting. Early Child Res Quart. (2012) 27:696–707. 10.1016/j.ecresq.2011.03.001 23166405 PMC3499624

[46] Choi JK Kelley MS Wang D. Neighborhood characteristics, maternal parenting, and health and development of children from socioeconomically disadvantaged families. Am J Community Psychol. (2018) 62:476–91. 10.1002/ajcp.12276 30239989

[47] Cuellar J Jones DJ Sterrett E. Examining parenting in the neighborhood context: a review. J Child Fam Stud. (2015) 24:195–219. 10.1007/s10826-013-9826-y 26392738 PMC4573634

[48] Barajas-Gonzalez RG Brooks-Gunn J. Income, neighborhood stressors, and harsh parenting: test of moderation by ethnicity, age, and gender. J Fam Psychol. (2014) 28:855–66. 10.1037/a0038242 25383794

[49] Odgers CL Caspi A Russell MA Sampson RJ Arsenault L Moffitt TE. Supportive parenting mediates neighborhood socioeconomic disparities in children's antisocial behavior from ages 5 to 12. Dev Psychopathol. (2012) 24:705–21. 10.1017/S0954579412000326 22781850 PMC3551477

[50] Dallaire DH Weinraub M. The stability of parenting behaviors over the first 6 years of life. Early Child Res Q. (2005) 20:201–19. 10.1016/j.ecresq.2005.04.00823889012

[51] Vallotton CD Mastergeorge AM Foster T Decker KB Ayoub C. Parenting supports for early vocabulary development: specific effects of sensitivity and stimulation through infancy. Infancy. (2017) 22:78–107. 10.1111/infa.12147 28111526 PMC5240633

[52] Farrant BM Devine TA Maybery MT Fletcher J. Empathy, perspective taking and prosocial behavior: the importance of parenting practices. Infant Child Dev. (2011) 21:175–88. 10.1002/icd.740

[53] Williams KE Berthelsen D. The development of prosocial behaviour in early childhood: contributions of early parenting and self-regulation. Int J Early Childh. (2017) 49:73–94. 10.1007/s13158-017-0185-5

[54] Healy KL Sanders MR Iyer A. Facilitative parenting and children's social, emotional, and behavioral adjustment. J Child Fam Stud. (2015) 24:1762–79. 10.1007/s10826-014-9980-x

[55] Lugo-Gil J Tamis-LeMonda CS. Family resources and parenting quality: links to children's cognitive development across the first 3 years. Child Dev. (2008) 79:1065–85. 10.1111/j.1467-8624.2008.01176.x 18717907

[56] Landry SH Smith KE Swank PR Garcia Coll C. Responsive parenting: establishing early foundations for social, communication, and independent problem-solving skills. Dev Psychol. (2006) 42:627–42. 10.1037/0012-1649.42.4.627 16802896

[57] Madigan S Prime H Graham SA Rodrigues M Anderson N Khoury J. Parenting behavior and child language: a meta-analysis. Pediatrics. (2019) 144:e20183556. 10.1542/peds.2018-3556 31551396

[58] Gross HE Shaw DS Moilanen KL Dishion TJ Wilson MN. Reciprocal models of child behavior and depressive symptoms in mothers and fathers in a sample of children at risk for early conduct problems. J Fam Psychol. (2008) 22:742–51. 10.1037/a0013514 18855510 PMC2710142

[59] Newton EK Laible D Carlo G Steele JS McGinley M. Do sensitive parents foster kind children, or vice versa? Bidirectional influences between children's prosocial behavior and parental sensitivity. Dev Psychol. (2014) 50:1808–16. 10.1037/a0036495 24708456

[60] Yan N Ansari A Fiese BH. Child adjustment and parent functioning: considering the role of child-driven effects. J Fam Psychol. (2016) 30:297–308. 10.1037/fam0000180 26866838 PMC4816670

[61] Burke JD Pardini DA Loeber R. Reciprocal relationships between parenting behavior and disruptive psychopathology from childhood through adolescence. J Abnorm Child Psychol. (2008) 36:679–92. 10.1007/s10802-008-9219-7 18286366 PMC2976977

[62] Calvete E Orue I Gamez-Guadix M Bushman BJ. Predictors of child-to-parent aggression: a 3-year longitudinal study. Dev Psychol. (2015) 51:663–76. 10.1037/a0039092 25822895

[63] Glatz T Stattin H Kerr M. Parents' reactions to youths' hyperactivity, impulsivity, and attention problems. J Abnorm Child Psychol. (2011) 39:1125–35. 10.1007/s10802-011-9541-3 21748550

[64] Humeniuk E Tarkowski Z. Parents' reactions to children's stuttering and style of coping with stress. J Fluency Disord. (2016) 49:51–60. 10.1016/j.jfludis.2016.08.002 27638192

[65] Davis M West K Bilms J Morelen D Suveg C. A systematic review of parent-child synchrony: it is more than skin deep. Dev Psychobiol. (2018) 60:674–91. 10.1002/dev.21743 29900545

[66] Ayoub M Briley DA Grotzinger A Patterson MW Engelhardt LE Tackett JL. Genetic and environmental associations between child personality and parenting. Soc Psychol Personal Sci. (2018) 10:711–21. 10.1177/1948550618784890 31807233 PMC6893882

[67] Steele H Bate J Steele M Dube SR Danskin K Knafo H. Adverse childhood experiences, poverty, and parenting stress. Can J Behav Sci. (2016) 48:32–8. 10.1037/cbs000003432804295

[68] Pastorelli C Lansford JE Kanacri BPL Malone PS Giunta LD Bacchini D. Positive parenting and children's prosocial behavior in eight countries. J Child Psychol Psychiatry. (2016) 57:824–34. 10.1111/jcpp.12477 26511201 PMC4848190

[69] Yan N Ansari A Wang Y. Intrusive parenting and child externalizing behaviors across childhood: the antecedents and consequences of child-driven effects. J Fam Psychol. (2019) 33:661–70. 10.1037/fam0000551 31259567

[70] Rothenberg WA Lansford JE Alampay LP Al-Hassan SM Bacchini D Bonrstein MH. Examining effects of mother and father warmth and control on child externalizing and internalizing problems from age 8 to 13 in nine countries. Dev Psychopathol. (2019) 32:1113–37. 10.1017/S0954579419001214 31865926 PMC7308194

[71] Lansford JE Criss MM Laird RD Shaw DS Pettit GS Bates JE. Reciprocal relations between parents' physical discipline and children's externalizing behavior during middle childhood and adolescence. Dev Psychopathol. (2011) 23:225–38. 10.1017/S0954579410000751 21262050 PMC3050569

[72] Yan N Ansari A Peng P. Reconsidering the relation between parental functioning and child externalizing behaviors: a meta-analysis on child-driven effects. J Fam Psychol. (2021) 35:225–35. 10.1037/fam0000805 33104378

[73] Reichman NE Teitler JO Garfinkel I McLanahan SS. Fragile families: sample and design. Child Youth Serv Rev. (2001) 23:303–26. 10.1016/S0190-7409(01)00141-4

[74] Hogan AE Scott KG Bauer CR. The Adaptive Social Behavior Inventory (ASBI): a new assessment of social competence in high-risk three-year-olds. J Psychoeduc Assess. (1992) 10:230–9. 10.1177/073428299201000303

[75] Dunn LM. Examiner's Manual for the Peabody Picture Vocabulary Test. 3rd ed. Circle Pines, MN: American Guidance Services, Inc. (1997). 10.1037/t15145-000

[76] Caldwell M Bradley RH. The Home Observation for Measurement of the Environment. Little Rock: University of Arkansas (1984).

[77] Rutter M. Protective factors in children's responses to stress and disadvantage. Ann Acad Med Singapore. (1979) 8:324–38. 547874

[78] Evans GW Li D Whipple SS. Cumulative risk and child development. Psychol Bull. (2013) 139:1342. 10.1037/a0031808 23566018

[79] Doan SN Fuller-Rowell TE Evans GW. Cumulative risk and adolescent's internalizing and externalizing problems: the mediating roles of maternal responsiveness and self-regulation. Dev Psychol. (2012) 48:1529–39. 10.1037/a0027815 22486443

[80] Center for Research on Child Wellbeing. Data User's Guide for the Nine-Year Follow-Up Wave of the Fragile Families Child Wellbeing Study. (2011). Available online at: http://www.fragilefamilies.princeton.edu/documentation/year9/year9wave_ff_public.pdf. (accessed May 27, 2020). Princeton University Bendheim-Thoman Center for Research on Child Wellbeing

[81] Muthén LK Muthén BO. Mplus: Statistical Analysis With Latent Variables: User's Guide (Version 8). Los Angeles, CA: Muthén & Muthén (2017).

[82] Byrne BM. Structural Equation Modeling With Amos: Basic Concepts, Applications, and Programming. Mahwah, NJ: Routledge (2001).

[83] Hu LT Bentler PM. Cutoff criteria for fit indexes in covariance structure analysis: conventional criteria versus new alternatives. Struct Eq Model. (1999) 6:1–55. 10.1080/10705519909540118

[84] Enders CK Bandalos DL. The relative performance of full information maximum likelihood estimation for missing data in structural equation models. Struct Equ Model. (2001) 8:430–57. 10.1207/S15328007SEM0803_5

[85] Thelen E Smith LB. Dynamic systems theories. In: Damon W Lerner RM editors, Handbook of Child Psychology: Theoretical Models of Human Development, Vol. 1. 6th ed. Hoboken, NJ: Wiley (2006). p. 258–312.

[86] Hollenstein T. Twenty years of dynamic systems approaches to development: significant contributions, challenges, and future directions. Child Dev Perspect. (2011) 5:256–9. 10.1111/j.1750-8606.2011.00210.x

[87] van Geert P. The contribution of complex dynamic systems to development. Child Dev Perspect. (2011) 4:273–8. 10.1111/j.1750-8606.2011.00197.x

[88] Ziv Y. Social information processing patterns, social skills, and school readiness in preschool children. J Exp Child Psychol. (2013) 114:306–20. 10.1016/j.jecp.2012.08.009 23046690 PMC3508340

[89] Beck L Kumschick IR Eid M Klann-Delius G. Relationship between language competence and emotional competence in middle childhood. Emotion. (2012) 12:503–14. 10.1037/a0026320 22148995

[90] Justice LM Jiang H Logan JAR Schmitt MB. Predictors of language gains among school-age children with language impairment in the public schools. J Speech Lang Hear Res. (2017) 6:1–16. 10.1044/2016_JSLHR-L-16-0026 28549355

[91] Kuhnert RL Begeer S Fink E de Rosnay M. Gender-differentiated effects of theory of mind, emotion understanding, and social preference on prosocial behavior development: a longitudinal study. J Exp Child Psychol. (2017) 154:13–27. 10.1016/j.jecp.2016.10.001 27780091

[92] Slot PL Bleses D Jensen P. Infants' and toddlers' language, math, and socio-emotional development: evidence for reciprocal relations and differential gender and age effects. Front Psychol. (2020) 23:580297. 10.3389/fpsyg.2020.580297 33329234 PMC7732521

[93] Montroy JJ Bowles RP Skibbe LE McClelland MM Morrison FJ. The development of self-regulation across early childhood. Dev Psychol. (2016) 52:1744–62. 10.1037/dev0000159 27709999 PMC5123795

[94] Veijalainen J Reunamo J Heikkilä M. Early gender differences in emotional expressions and self-regulation in settings of early childhood education and care. Early Child Dev Care. (2021) 191:173–86. 10.1080/03004430.2019.1611045

[95] Cabrera NJ Volling BL Barr R. Fathers are parents, too! Widening the lens on parenting for children's development. Child Dev Perspectiv. (2018) 12:152–7. 10.1111/cdep.12275

